# Supplementary material for: Evaluating Yield, Nutritional Quality, and Environmental Impact of Quinoa Straws across Mediterranean Water Environments
Source: Plants (Basel). 2024 Mar 7;13(6):751. doi: 10.3390/plants13060751 (PMC10974331; doi:10.3390/plants13060751)
Supplement: Supplementary file 1 [file plants-13-00751-s001.zip › plants-2796166-supplementary.pdf]

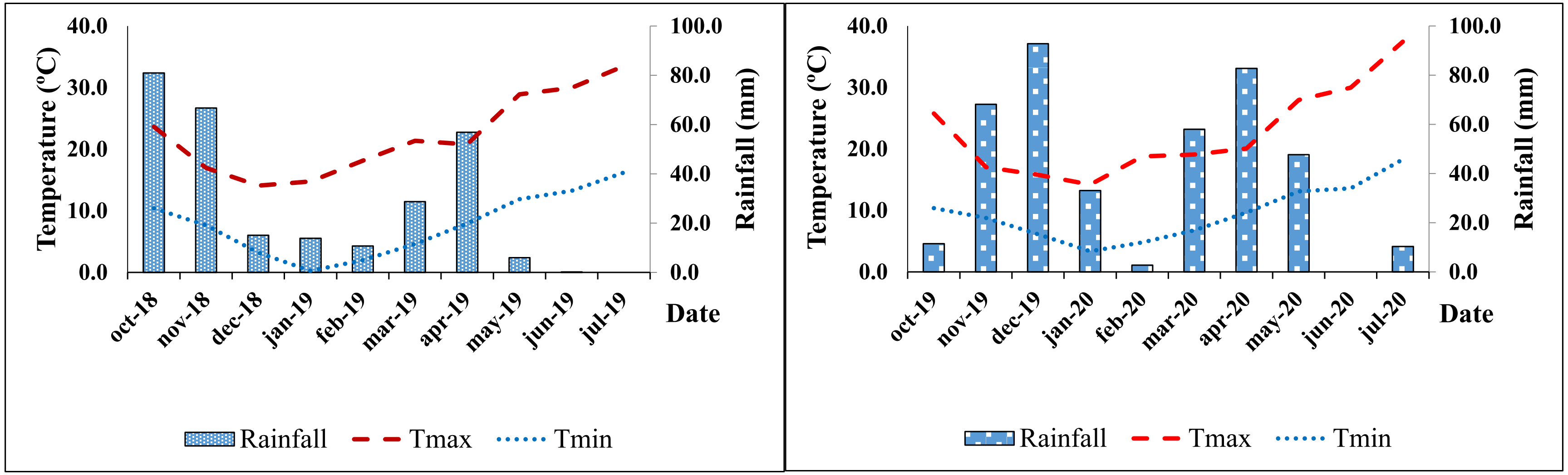

a)

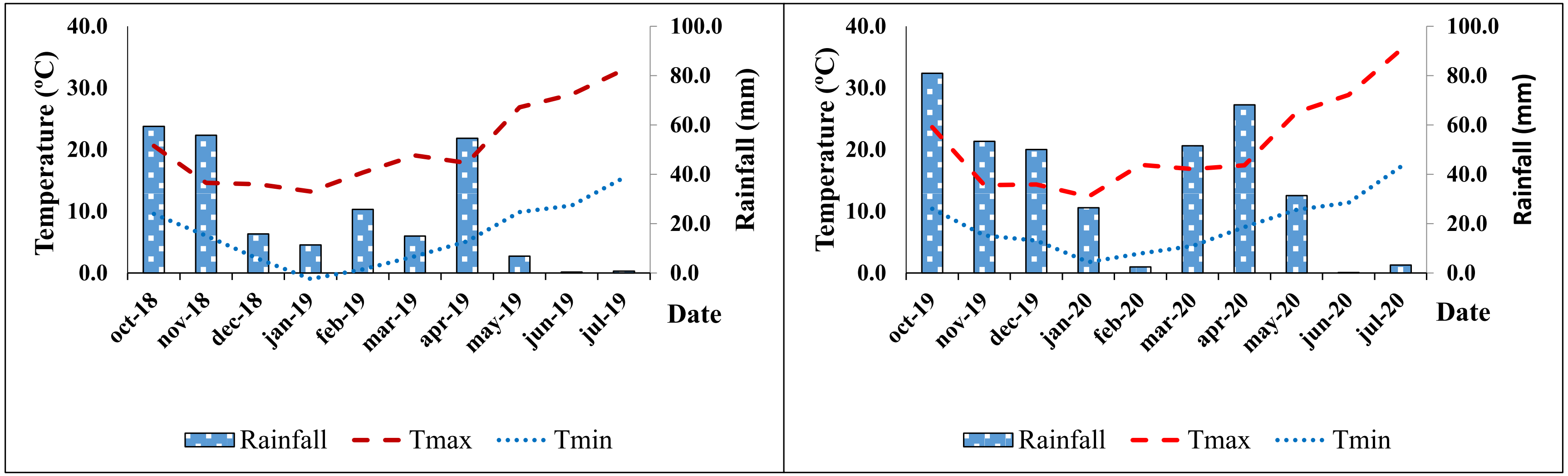

b)

Supplementary Figure S1. Monthly rainfall and maximum (Tmax) and minimum (Tmin) mean temperature registered at a) La Orden farm and b) Maguilla during the 2019 (left) and 2020 (right) seasons

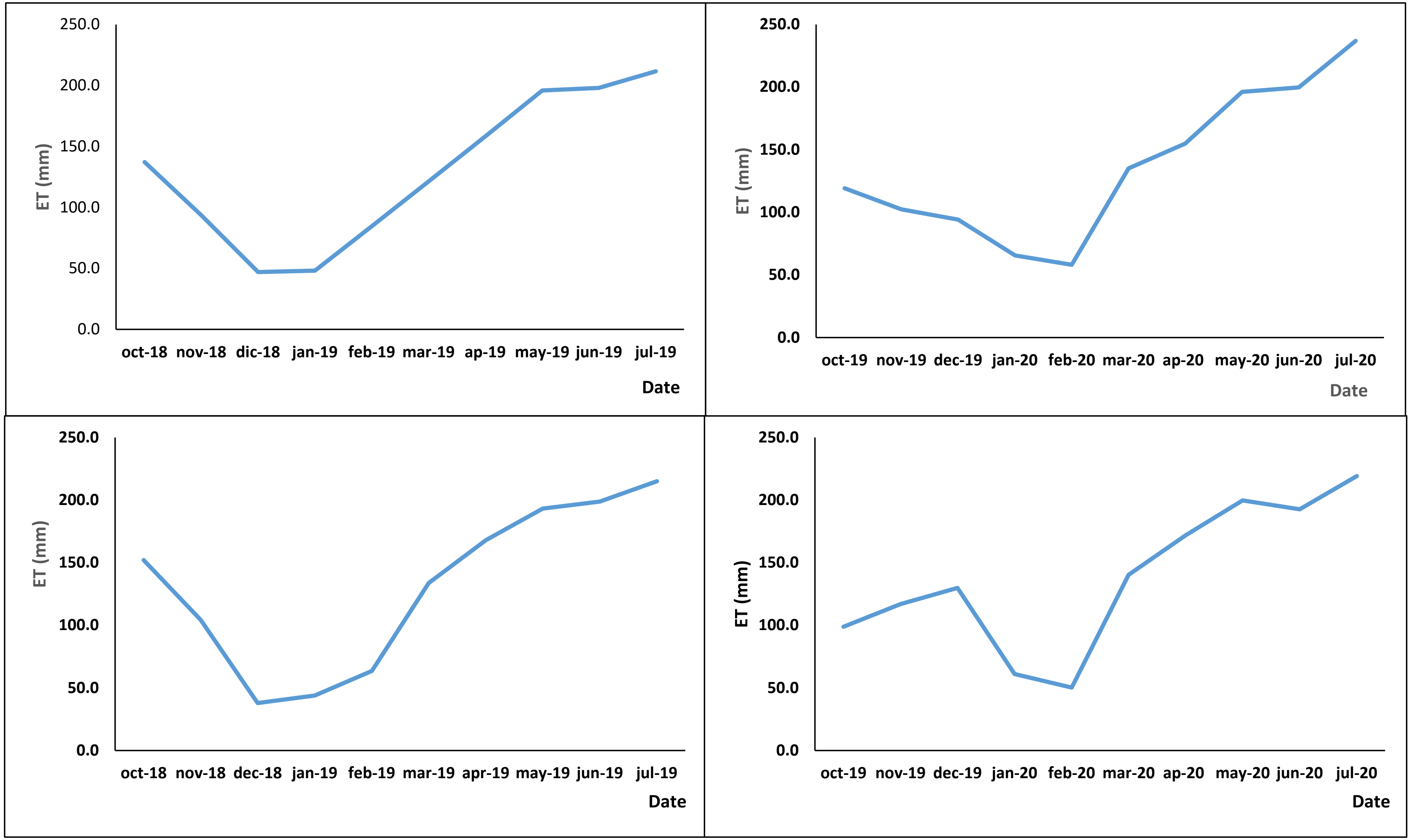

Supplementary Figure S2. **Monthly evapotranspiration (ET) at a) La Orden farm and b) Maguilla during the 2019 (left) and 2020 (right) experiments.** ET values were calculated using the following information: La Orden (Field capacity: 17.1% w/w; Wilting point: 7.9% w/w; Bulk density: 1.61 t/m³; Depth >1 m) and Maguilla (Field capacity: 37.0% w/w; Wilting point: 24.6% w/w; Bulk density: 1.45 t/m³; Depth >1 m).

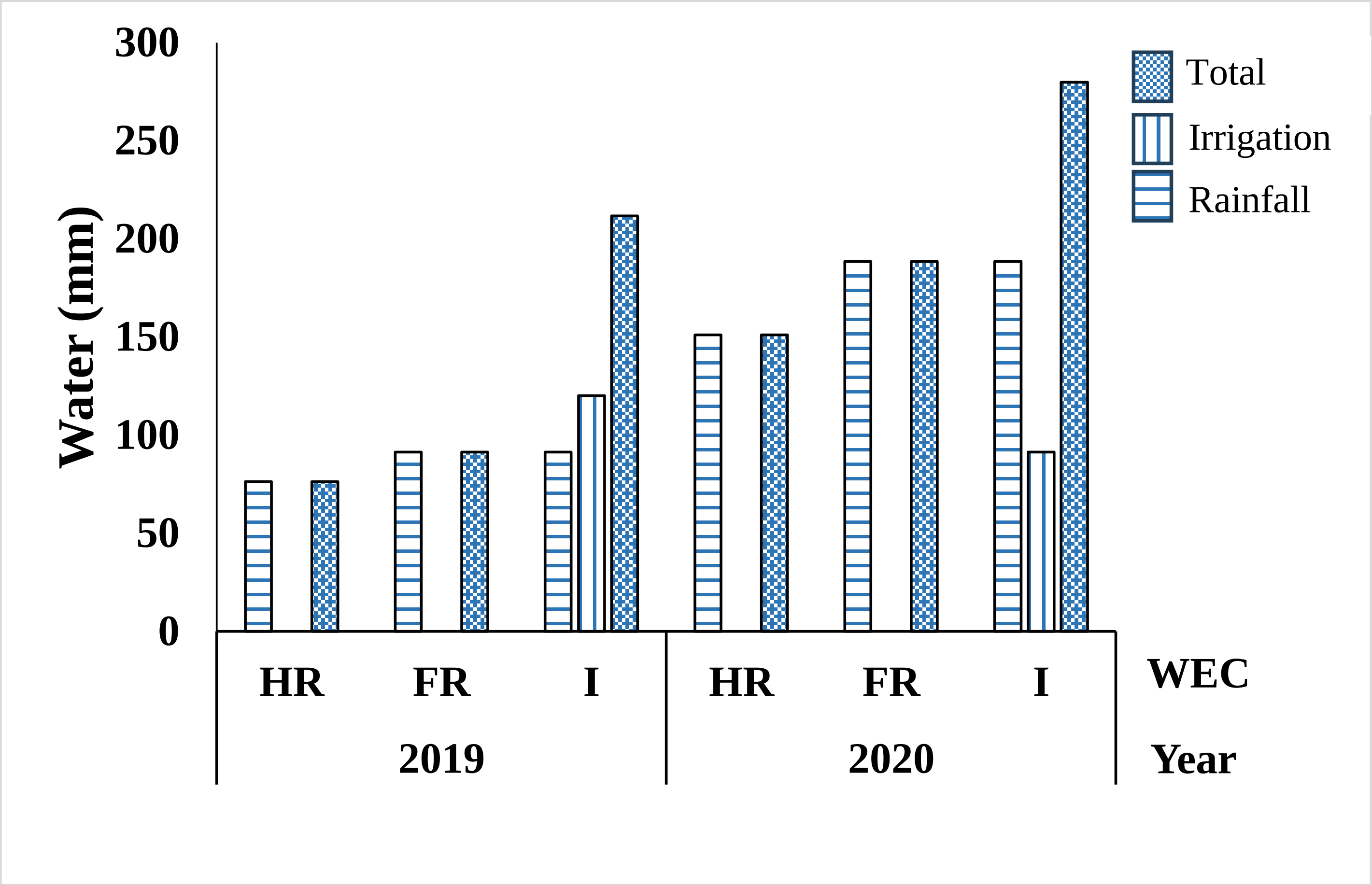

Supplementary Figure S3. **Water input in each water environmental condition (WEC).** Under rainfed conditions (HR or FR), the water input was due to precipitations, and under irrigated conditions (I), the water input was the sum of the water due to precipitations and irrigation. Precipitations (rainfall) in the graph correspond those in record from March to June (growing season in La Orden farm (FR and I) and Maguilla (HR) during the 2019 and 2020 seasons.

Table S1. Straw yield (kg ha^{-1}) and harvest index (HI) of three quinoa varieties (V) grown under three water environmental conditions (WEC) during two consecutive years (Y) according to treatments interactions.

Interaction	Straw yield (kg ha^{-1})	HI
**Y x WEC**		
2019 x I	2805 ab	0.43 a
2019 x FR	1188 d	0.44 a
2019 x HR	2094 bc	0.28 b
2020 x I	2555 abc	0.44 a
2020 x FR	3050 a	0.42 a
2020 x HR	1762 cd	0.41 a
**Y x V**		
2019 x P	2468 a	0.35
2019 x M	2214 a	0.37
2019 x T	1405 b	0.44
2020 x P	2487 a	0.42
2020 x M	2459 a	0.43
2020 x T	2420 a	0.42
**WEC x V**		
I x P	3096 a	0.42
I x M	2770 ab	0.43
I x T	2175 abc	0.45
FR x P	2193 abc	0.43
FR x M	2176 abc	0.42
FR x T	1987 bc	0.45
HR x P	2144 bc	0.32
HR x M	2064 bc	0.34
HR x T	1576 c	0.39

Different lowercase letters within the same column indicate significant differences at $p < 0.05$ according to Tukey's test. n.s.: not significant; significant at *p<0.05; **p <0.01 and *** p < 0.001. I: irrigated. FR: fresh rainfed. HR hard rainfed; P: Pasto. M: Marisma. T: Titicaca

Table S2. Content of ash, protein, minerals and fibre, and relative feed value (RFV) of the straw of three quinoa varieties (Pasto, Marisma, and Titicaca) grown under three environmental conditions (I, FR and HR) during two consecutive years (2019, 2020).

Treatment	Ash (%)	CP (%)	CF (%)	P (%)	K (%)	Ca (%)	Mg (%)	GF (%)	NDF (%)	ADF (%)	ADL (%)	Hem. (%)	Cell. (%)	RFV
**Y x WEC**														
2019 x I	13.9	15.9 a	1.75	0.36 a	5.74 ab	2.24 ab	1.21 a	20.6	34.2 c	23.5	4.7	10.7	18.8 c	195.2 a
2019 x FR	14.9	14.2 a	2.11	0.22 b	6.12 ab	2.74 a	1.14 a	21.4	32.3 c	21.7	3.9	10.6	17.7 c	216.4 a
2019 x HR	19.2	9.1 b	2.17	0.07 c	6.39 a	1.97 b	0.43 c	25.9	43.8 b	30.4	4.4	13.4	26.0 b	138.9 b
2020 x I	14.2	15.2 a	0.90	0.25 ab	4.66 c	1.92 b	0.86 b	30.6	49.1 a	35.4	5.6	13.7	29.9 ab	116.7 bc
2020 x FR	13.7	14.4 a	1.08	0.27 ab	5.53 b	1.79 b	0.74 b	30.2	47.0 ab	34.2	4.9	12.8	29.3 ab	124.5 bc
2020 x HR	18.8	9.5 b	0.89	0.17 bc	6.11 ab	2.68 a	0.44 c	33.3	51.3 a	37.7	5.1	13.6	32.6 a	108.7 c
**Y x V**														
2019 x P	15.1	11.0	2.20	0.18 ab	6.04 a	2.44	0.87 ab	23.5	38.3	26.5	4.7	11.8	21.8	169.2
2019 x M	16.5	13.8	2.07	0.22 ab	5.71 ab	2.38	1.01 a	23.5	35.7	24.5	4.3	11.2	20.2	189.2
2019 x T	16.5	14.5	1.77	0.25 ab	6.51 a	2.13	0.91 ab	20.8	36.3	24.6	4.1	11.7	20.5	192.2
2020 x P	14.0	11.3	1.00	0.25 ab	5.08 b	2.11	0.66 ab	32.1	50.3	36.9	5.3	13.4	31.5	112.4
2020 x M	16.3	13.1	0.92	0.29 a	5.63 ab	2.29	0.78 ab	30.5	47.7	34.5	5.0	13.2	29.5	122.6
2020 x T	16.4	14.6	0.96	0.16 b	5.59 ab	1.99	0.59 b	31.5	49.4	35.9	5.2	13.5	30.8	114.9
**WEC x V**														
I x P	11.1 d	12.7 c	1.55	0.31	4.91	1.95	0.92	27.2	44.7	32.5 ab	5.6	12.3	26.9 ab	136.7
I x M	15.5 c	16.7 ab	1.28	0.33	4.91	2.21	1.15	26.5	41.2	28.8 ab	5.0	12.4	23.8 ab	157.8
I x T	15.6 bc	17.2 a	1.16	0.29	5.80	2.09	1.05	23.0	39.1	27.2 ab	4.8	11.9	22.4 ab	173.4
FR x P	13.6 cd	11.5 cd	1.59	0.20	5.83	2.49	0.89	26.5	40.3	28.8 ab	4.5	11.5	24.3 ab	161.7
FR x M	14.3 c	14.1 bc	1.83	0.30	5.86	2.39	1.16	25.3	36.8	25.8 b	4.2	11.0	21.5 b	184.1
FR x T	15.0 c	17.3 a	1.37	0.25	5.79	1.92	0.88	25.6	41.8	29.2 ab	4.5	12.6	24.8 ab	165.5
HR x P	18.9 a	9.3 d	1.66	0.13	5.94	2.39	0.49	29.6	47.8	33.8 ab	4.9	13.9	29.0 ab	123.9
HR x M	19.3 a	9.5 d	1.37	0.14	6.25	2.41	0.59	29.3	47.1	34.0 ab	4.7	13.2	29.2 ab	125.9
HR x T	18.8 ab	9.1 d	1.56	0.09	6.56	2.17	0.33	29.8	47.2	34.4 a	4.6	13.4	29.8 a	121.7

Different lowercase letters within the same column indicate significant difference at $p < 0.05$ according to Tukey's test.. n.s.: not significant; significant at *p<0.05; **p <0.01 and *** p < 0.001. I: irrigated. FR: fresh rainfed. HR hard rainfed

Table S3. Results of interactions between treatments for the minor fatty acids contents in seeds harvested from three quinoa varieties (Pasto, Marisma, and Titicaca) grown under three different environmental conditions (I, FR, HR) during two consecutive years (2019, 2020).

Treatment	NU (kg ha^{-1}	PU (kg ha^{-1})	KU (kg ha^{-1})	CaU (kg ha^{-1})	MgU (kg ha^{-1})	NUtE (kg/kg)	PUtE (kg/kg)	KUtE (kg/kg)	CaUtE (kg/kg)	MgUtE (kg/kg)	NHI	PHI	KHI	CaHI	MgHI
**Y x WEC**															
2019 x I	118.2 a	17.0 a	175.7 ab	63.5 a	37.6 a	17.2	124.8 b	11.8 a	33.2	56.1	0.42 b	0.41	0.11 a	0.03	0.12
2019 x FR	49.0 b	5.3 b	83.1 d	35.2 c	15.4 c	19.1	191.8 b	11.9 a	30.5	66.9	0.47 ab	0.56	0.13 ab	0.05	0.14
2019 x HR	51.7 b	3.3 b	141.6 bc	42.2 bc	10.4 c	16.1	264.8 a	5.7 b	20.6	90.6	0.41 b	0.54	0.06 c	0.02	0.14
2020 x I	113.5 a	14.6 a	142.5 bc	51.9 abc	26.5 b	18.0	138.3 b	14.4 a	39.9	78.5	0.46 b	0.56	0.16 a	0.06	0.18
2020 x FR	125.0 a	18.5 a	195.9 a	57.1 ab	28.1 ab	18.3	123.7 b	11.7 a	41.6	83.0	0.44 b	0.56	0.14 ab	0.05	0.20
2020 x HR	62.0 b	7.9 b	175.7 ab	48.6 abc	10.5 c	20.1	165.6 b	10.3 a	26.3	104.9	0.57 a	0.62	0.12 ab	0.03	0.24
**Y x V**															
2019 x P	79.0 ab	10.7	160.8 ab	57.8 a	25.4 a	17.3	172.1	8.5	22.7	59.7	0.44	0.54	0.10	0.03	0.13
2019 x M	83.4 ab	9.2	138.0 ab	53.0 a	24.9 a	16.2	178.0	9.7	24.9	58.0	0.40	0.48	0.10	0.03	0.11
2019 x T	56.5 b	5.6	101.6 b	30.1 b	13.1 b	18.8	231.2	11.4	36.8	95.9	0.46	0.49	0.10	0.04	0.15
2020 x P	91.9 ab	14.0	148.3 ab	53.1 a	21.1 ab	20.0	138.9	12.8	36.8	93.4	0.51	0.55	0.15	0.04	0.20
2020 x M	100.8 ab	15.7	126.3 a	57.4 a	24.6 a	19.0	126.0	11.7	34.2	85.4	0.49	0.54	0.15	0.04	0.20
2020 x T	107.7 a	11.3	149.8 ab	47.1 ab	19.4 ab	17.4	162.6	11.9	37.0	107.7	0.46	0.60	0.12	0.05	0.22
**WEC x V**															
I x P	115.0	18.8	180.8	63.8	34.6	19.2	122.4	13.1	36.2	71.0 b	0.46	0.49	0.15	0.05	0.16
I x M	127.9	16.8	159.2	63.5	36.5	16.5	123.6	13.3	34.7	60.7 b	0.43	0.47	0.15	0.04	0.14
I x T	104.5	11.8	137.3	45.7	25.1	17.2	148.6	12.8	38.8	70.2 b	0.43	0.50	0.11	0.04	0.14
FR x P	80.8	12.2	144.5	51.0	23.0	20.6	161.8	11.5	33.9	76.1 b	0.50	0.62	0.14	0.04	0.18
FR x M	89.0	13.9	148.0	51.6	25.4	18.2	143.6	10.9	31.4	62.7 b	0.45	0.51	0.14	0.05	0.15
FR x T	91.3	9.5	126.0	35.9	17.0	17.3	167.8	13.1	42.9	86.2 b	0.42	0.55	0.13	0.06	0.19
HR x P	60.7	6.1	138.5	51.7	13.2	16.1	182.4	7.4	19.0	82.6 b	0.46	0.52	0.09	0.02	0.16
HR x M	59.4	6.6	143.2	50.4	12.4	18.2	188.9	7.9	22.4	91.9 b	0.47	0.56	0.10	0.03	0.18
HR x T	50.5	4.1	113.8	34.2	6.7	19.9	204.1	9.1	29.0	148.9 a	0.54	0.66	0.09	0.03	0.23

Different lowercase letters within the same column indicate significant difference at p< 0.05 according to Tukey's test. n.s.: not significant; significant at *p<0.05; **p <0.01 and *** p < 0.001. I: irrigated. FR: fresh rainfed. HR: hard rainfed. P: Pasto; M: Marisma; T: Titicaca.

Table S4. PCA rotated component matrix.

Influence	Component 1		Component 2		Component 3	
Positive	Cellulose	0.985	KUtE	0.96	Straw yield	0.96
	ADF	0.978	HI	0.938	KU	0.904
	NDF	0.977	KHI	0.9	NU	0.853
	GF	0.957	CaUtE	0.833	CaU	0.837
	Hemicellulose	0.855	CaHI	0.796	MgU	0.813
	MgHI	0.831	P	0.721	PU	0.778
	MgUtE	0.731	CP	0.601	ADL	0.529
	PHI	0.628	PU	0.542		
	ADL	0.619	Mg	0.525		
	NHI	0.489	NUtE	0.517		
			NU	0.411		
Negative	RFV	-0.956	Ash	-0.698	NHI	-0.601
	Mg	-0.814	PUtE	-0.628	K	-0.579
	CF	-0.698	K	-0.535	Ca	-0.573
	CP	-0.460	CF	-0.525	NUtE	-0.547
	P	-0.406			PUtE	-0.534
					MgUtE	-0.496
					PHI	-0.467

Table S5. t-Student comparisons. **A**. Year 2019. **B**. Year 2020. Tables containing the p-value of the comparison between straw and seed mineral content. P-adjusted calculated by applying a BH correction in all the comparisons performed for each mineral. Significant differences considering the p-adjusted are indicated with *.

A.

Environmental conditions	Cultivar	Mineral	p.value	p.adjusted	Significance
HR	Pasto	P	6.92687E-05	0.000623418	*
		K	0.003211811	0.004447123	*
		Ca	0.003672094	0.00647574	*
		Mg	0.00345344	0.01036032	*
	Marisma	P	0.004209938	0.015155777	*
		K	0.002123959	0.003475569	*
		Ca	0.001764432	0.006106872	*
		Mg	0.004927527	0.012670784	*
	Titicaca	P	0.038011005	0.052630622	
		K	0.002904883	0.004357325	*
		Ca	3.12764E-07	5.62975E-06	*
		Mg	0.007371433	0.016585724	*
FR	Pasto	P	0.00312432	0.01405944	*
		K	0.012849361	0.012849361	*
		Ca	0.007200339	0.009257579	*
		Mg	0.082172438	0.082172438	
	Marisma	P	0.573700988	0.607448105	
		K	0.003672236	0.004721446	*
		Ca	0.005067068	0.00701594	*
		Mg	0.013297703	0.019946555	*
	Titicaca	P	0.663917607	0.663917607	
		K	5.79845E-05	0.000347907	*
		Ca	0.002683746	0.00647574	*
		Mg	0.023056029	0.027667235	*
I	Pasto	P	0.233227361	0.262380781	
		K	0.000677818	0.001742961	*
		Ca	4.02173E-06	3.61956E-05	*
		Mg	0.003336406	0.01036032	*
	Marisma	P	0.226427032	0.262380781	
		K	0.000226007	0.000760788	*
		Ca	0.003489547	0.00647574	*
		Mg	0.012813189	0.019946555	*
	Titicaca	P	0.030318142	0.049611505	*
		K	0.000031189	0.000280701	*
		Ca	0.008017493	0.009620992	*
		Mg	0.010103445	0.019946555	*
HR	Pasto	P	0.036717981	0.052630622	
		K	0.000847265	0.001906346	*
		Ca	0.020369281	0.021567474	*
		Mg	0.002626093	0.01036032	*
	Marisma	P	0.021575353	0.038835635	*
		K	0.001534285	0.002761713	*
		Ca	0.027549119	0.027549119	*
		Mg	0.029165787	0.030881422	*
	Titicaca	P	0.001929296	0.011575776	*
		K	0.004843933	0.005449425	*
		Ca	0.001174069	0.006106872	*
		Mg	0.022312565	0.027667235	*

B.

Environmental conditions	Cultivar	Mineral	p.value	p.adjusted	Significance
HR	Pasto	P	0.036717981	0.052630622	
		K	0.000847265	0.001906346	*
		Ca	0.020369281	0.021567474	*
		Mg	0.002626093	0.01036032	*
	Marisma	P	0.021575353	0.038835635	*
		K	0.001534285	0.002761713	*
		Ca	0.027549119	0.027549119	*
		Mg	0.029165787	0.030881422	*
	Titicaca	P	0.001929296	0.011575776	*
		K	0.004843933	0.005449425	*
		Ca	0.001174069	0.006106872	*
		Mg	0.022312565	0.027667235	*
FR	Pasto	P	0.008229341	0.018618651	*
		K	2.87634E-06	5.17741E-05	*
		Ca	0.010781513	0.012129202	*
		Mg	0.016064931	0.022243751	*
	Marisma	P	0.048708065	0.062624655	
		K	0.004754641	0.005449425	*
		Ca	0.003627399	0.00647574	*
		Mg	0.001058914	0.009530226	*
	Titicaca	P	5.88852E-05	0.000623418	*
		K	9.44204E-05	0.000424892	*
		Ca	0.004103277	0.00647574	*
		Mg	0.024718877	0.027808737	*
I	Pasto	P	0.005657994	0.016973982	*
		K	0.006723923	0.007119448	*
		Ca	0.001791979	0.006106872	*
		Mg	0.002186824	0.01036032	*
	Marisma	P	0.014391036	0.028782072	*
		K	0.000253596	0.000760788	*
		Ca	0.00431716	0.00647574	*
		Mg	0.012396747	0.019946555	*
	Titicaca	P	0.008274956	0.018618651	*
		K	0.001453019	0.002761713	*
		Ca	0.002035624	0.006106872	*
		Mg	9.61807E-08	1.73125E-06	*

SUPPLEMENTARY FILE 1

	Straw yield	HI	Ash	CP	CF	P
Straw yield	1.000	-0.317	-0.194	-0.134	0.019	0.177
HI	-0.317	1.000	-0.482	0.729	-0.507	0.507
Ash	-0.194	-0.482	1.000	-0.395	0.188	-0.518
CP	-0.134	0.729	-0.395	1.000	-0.503	0.712
CF	0.019	-0.507	0.188	-0.503	1.000	-0.514
P	0.177	0.507	-0.518	0.712	-0.514	1.000
K	-0.475	-0.082	0.483	-0.292	-0.023	-0.264
Ca	-0.339	0.341	-0.156	0.223	0.094	0.115
Mg	-0.058	0.621	-0.706	0.767	-0.303	0.807
GF	0.279	-0.584	0.527	-0.644	0.470	-0.793
NDF	0.353	-0.641	0.514	-0.712	0.308	-0.673
ADF	0.413	-0.609	0.407	-0.692	0.309	-0.658
ADL	0.739	-0.294	-0.165	-0.144	0.175	0.117
Hemicellulose	0.066	-0.497	0.613	-0.508	0.196	-0.472
Cellulose	0.328	-0.604	0.459	-0.716	0.302	-0.718
RFV	-0.456	0.663	-0.461	0.704	-0.253	0.625
NU	0.834	0.162	-0.389	0.389	-0.273	0.493
PU	0.804	0.196	-0.559	0.375	-0.270	0.645
KU	0.981	-0.338	-0.133	-0.207	0.003	0.132
CaU	0.922	-0.202	-0.269	-0.022	0.037	0.243
MgU	0.786	0.147	-0.609	0.381	-0.201	0.613
NUtE	-0.285	0.713	-0.306	0.070	-0.241	0.089
PUtE	-0.320	-0.070	0.457	-0.488	0.119	-0.710
KUtE	-0.238	0.963	-0.491	0.777	-0.523	0.553
CaUtE	-0.221	0.874	-0.351	0.640	-0.608	0.487
MgUtE	-0.225	0.141	0.403	-0.297	-0.184	-0.443
NHI	-0.356	0.686	-0.179	0.049	-0.191	-0.066
PHI	-0.279	0.061	0.206	-0.342	0.322	-0.777
KHI	-0.193	0.850	-0.610	0.615	-0.330	0.408
CaHI	-0.347	0.581	-0.309	0.400	-0.029	0.095
MgHI	-0.232	0.317	0.200	-0.147	-0.081	-0.424

K	Ca	Mg	GF	NDF	ADF	ADL
-0.475	-0.339	-0.058	0.279	0.353	0.413	0.739
-0.082	0.341	0.621	-0.584	-0.641	-0.609	-0.294
0.483	-0.156	-0.706	0.527	0.514	0.407	-0.165
-0.292	0.223	0.767	-0.644	-0.712	-0.692	-0.144
-0.023	0.094	-0.303	0.470	0.308	0.309	0.175
-0.264	0.115	0.807	-0.793	-0.673	-0.658	0.117
1.000	0.095	-0.450	-0.042	0.104	0.031	-0.282
0.095	1.000	0.447	-0.318	-0.670	-0.694	-0.513
-0.450	0.447	1.000	-0.788	-0.883	-0.847	-0.143
-0.042	-0.318	-0.788	1.000	0.808	0.830	0.202
0.104	-0.670	-0.883	0.808	1.000	0.966	0.407
0.031	-0.694	-0.847	0.830	0.966	1.000	0.497
-0.282	-0.513	-0.143	0.202	0.407	0.497	1.000
0.260	-0.364	-0.662	0.457	0.730	0.529	0.023
0.074	-0.664	-0.881	0.854	0.969	0.992	0.380
-0.061	0.653	0.856	-0.794	-0.968	-0.955	-0.502
-0.566	-0.140	0.323	-0.040	-0.062	0.017	0.558
-0.513	-0.074	0.458	-0.215	-0.188	-0.098	0.591
-0.308	-0.345	-0.151	0.302	0.398	0.454	0.735
-0.494	0.040	0.138	0.156	0.097	0.143	0.560
-0.651	0.019	0.552	-0.237	-0.251	-0.175	0.506
0.138	0.221	0.177	-0.243	-0.209	-0.200	-0.270
0.298	-0.237	-0.677	0.541	0.541	0.525	-0.286
-0.264	0.266	0.671	-0.570	-0.617	-0.594	-0.256
-0.045	-0.116	0.400	-0.491	-0.345	-0.324	-0.111
0.465	-0.253	-0.607	0.366	0.456	0.436	-0.229
0.189	0.213	0.016	-0.082	-0.127	-0.097	-0.282
0.208	0.171	-0.486	0.578	0.356	0.371	-0.213
-0.397	0.371	0.661	-0.382	-0.589	-0.528	-0.200
-0.022	0.113	0.293	-0.169	-0.207	-0.192	-0.125
0.467	0.098	-0.433	0.312	0.271	0.253	-0.199

Hemicellulose	Cellulose	RFV	NU	PU	KU	CaU	
0.066	0.328	-0.456	0.834	0.804	0.981	0.922	
-0.497	-0.604	0.663	0.162	0.196	-0.338	-0.202	
0.613	0.459	-0.461	-0.389	-0.559	-0.133	-0.269	
-0.508	-0.716	0.704	0.389	0.375	-0.207	-0.022	
0.196	0.302	-0.253	-0.273	-0.270	0.003	0.037	
-0.472	-0.718	0.625	0.493	0.645	0.132	0.243	
0.260	0.074	-0.061	-0.566	-0.513	-0.308	-0.494	
-0.364	-0.664	0.653	-0.140	-0.074	-0.345	0.040	
-0.662	-0.881	0.856	0.323	0.458	-0.151	0.138	
0.457	0.854	-0.794	-0.040	-0.215	0.302	0.156	
0.730	0.969	-0.968	-0.062	-0.188	0.398	0.097	
0.529	0.992	-0.955	0.017	-0.098	0.454	0.143	
0.023	0.380	-0.502	0.558	0.591	0.735	0.560	
1.000	0.560	-0.653	-0.251	-0.361	0.107	-0.060	
0.560	1.000	-0.943	-0.065	-0.193	0.372	0.067	
-0.653	-0.943	1.000	-0.051	0.084	-0.503	-0.208	
-0.251	-0.065	-0.051	1.000	0.934	0.791	0.840	
-0.361	-0.193	0.084	0.934	1.000	0.772	0.824	
0.107	0.372	-0.503	0.791	0.772	1.000	0.891	
-0.060	0.067	-0.208	0.840	0.824	0.891	1.000	
-0.362	-0.263	0.148	0.909	0.957	0.718	0.858	
-0.158	-0.173	0.264	-0.131	-0.056	-0.247	-0.242	
0.389	0.603	-0.462	-0.475	-0.631	-0.280	-0.453	
-0.456	-0.594	0.639	0.234	0.248	-0.295	-0.143	
-0.276	-0.328	0.387	0.172	0.184	-0.230	-0.287	
0.345	0.499	-0.391	-0.263	-0.389	-0.140	-0.359	
-0.161	-0.061	0.167	-0.169	-0.168	-0.317	-0.327	
0.189	0.427	-0.294	-0.331	-0.449	-0.230	-0.236	
-0.538	-0.532	0.590	0.205	0.259	-0.259	-0.059	
-0.172	-0.186	0.344	-0.116	-0.032	-0.356	-0.307	
0.221	0.298	-0.205	-0.176	-0.242	-0.132	-0.219	

MgU	NUtE	PUtE	KUtE	CaUtE	MgUtE	NHI
0.786	-0.285	-0.320	-0.238	-0.221	-0.225	-0.356
0.147	0.713	-0.070	0.963	0.874	0.141	0.686
-0.609	-0.306	0.457	-0.491	-0.351	0.403	-0.179
0.381	0.070	-0.488	0.777	0.640	-0.297	0.049
-0.201	-0.241	0.119	-0.523	-0.608	-0.184	-0.191
0.613	0.089	-0.710	0.553	0.487	-0.443	-0.066
-0.651	0.138	0.298	-0.264	-0.045	0.465	0.189
0.019	0.221	-0.237	0.266	-0.116	-0.253	0.213
0.552	0.177	-0.677	0.671	0.400	-0.607	0.016
-0.237	-0.243	0.541	-0.570	-0.491	0.366	-0.082
-0.251	-0.209	0.541	-0.617	-0.345	0.456	-0.127
-0.175	-0.200	0.525	-0.594	-0.324	0.436	-0.097
0.506	-0.270	-0.286	-0.256	-0.111	-0.229	-0.282
-0.362	-0.158	0.389	-0.456	-0.276	0.345	-0.161
-0.263	-0.173	0.603	-0.594	-0.328	0.499	-0.061
0.148	0.264	-0.462	0.639	0.387	-0.391	0.167
0.909	-0.131	-0.475	0.234	0.172	-0.263	-0.169
0.957	-0.056	-0.631	0.248	0.184	-0.389	-0.168
0.718	-0.247	-0.280	-0.295	-0.230	-0.140	-0.317
0.858	-0.242	-0.453	-0.143	-0.287	-0.359	-0.327
1.000	-0.121	-0.669	0.227	0.071	-0.520	-0.258
-0.121	1.000	0.348	0.623	0.639	0.473	0.879
-0.669	0.348	1.000	-0.121	0.055	0.868	0.507
0.227	0.623	-0.121	1.000	0.887	0.041	0.583
0.071	0.639	0.055	0.887	1.000	0.298	0.617
-0.520	0.473	0.868	0.041	0.298	1.000	0.604
-0.258	0.879	0.507	0.583	0.617	0.604	1.000
-0.460	0.354	0.630	-0.051	-0.068	0.522	0.494
0.267	0.593	-0.155	0.885	0.682	-0.084	0.566
-0.059	0.428	0.020	0.537	0.521	0.099	0.414
-0.368	0.545	0.579	0.163	0.280	0.770	0.665

PHI	KHI	CaHI	MgHI
-0.279	-0.193	-0.347	-0.232
0.061	0.850	0.581	0.317
0.206	-0.610	-0.309	0.200
-0.342	0.615	0.400	-0.147
0.322	-0.330	-0.029	-0.081
-0.777	0.408	0.095	-0.424
0.208	-0.397	-0.022	0.467
0.171	0.371	0.113	0.098
-0.486	0.661	0.293	-0.433
0.578	-0.382	-0.169	0.312
0.356	-0.589	-0.207	0.271
0.371	-0.528	-0.192	0.253
-0.213	-0.200	-0.125	-0.199
0.189	-0.538	-0.172	0.221
0.427	-0.532	-0.186	0.298
-0.294	0.590	0.344	-0.205
-0.331	0.205	-0.116	-0.176
-0.449	0.259	-0.032	-0.242
-0.230	-0.259	-0.356	-0.132
-0.236	-0.059	-0.307	-0.219
-0.460	0.267	-0.059	-0.368
0.354	0.593	0.428	0.545
0.630	-0.155	0.020	0.579
-0.051	0.885	0.537	0.163
-0.068	0.682	0.521	0.280
0.522	-0.084	0.099	0.770
0.494	0.566	0.414	0.665
1.000	0.112	0.355	0.768
0.112	1.000	0.575	0.162
0.355	0.575	1.000	0.427
0.768	0.162	0.427	1.000

	Straw yield	HI	Ash	CP	CF	P
Straw yield	0.000	0.107	0.333	0.506	0.923	0.378
HI	0.107	0.000	0.011	0.000	0.007	0.007
Ash	0.333	0.011	0.000	0.042	0.348	0.006
CP	0.506	0.000	0.042	0.000	0.007	0.000
CF	0.923	0.007	0.348	0.007	0.000	0.006
P	0.378	0.007	0.006	0.000	0.006	0.000
K	0.012	0.684	0.011	0.139	0.909	0.184
Ca	0.084	0.082	0.436	0.263	0.641	0.568
Mg	0.773	0.001	0.000	0.000	0.124	0.000
GF	0.159	0.001	0.005	0.000	0.013	0.000
NDF	0.071	0.000	0.006	0.000	0.118	0.000
ADF	0.032	0.001	0.035	0.000	0.117	0.000
ADL	0.000	0.137	0.411	0.474	0.382	0.561
Hemicellulose	0.743	0.008	0.001	0.007	0.326	0.013
Cellulose	0.095	0.001	0.016	0.000	0.125	0.000
RFV	0.017	0.000	0.016	0.000	0.203	0.000
NU	0.000	0.419	0.045	0.045	0.169	0.009
PU	0.000	0.327	0.002	0.054	0.173	0.000
KU	0.000	0.085	0.508	0.300	0.986	0.513
CaU	0.000	0.312	0.175	0.914	0.856	0.223
MgU	0.000	0.463	0.001	0.050	0.315	0.001
NUtE	0.150	0.000	0.121	0.728	0.225	0.659
PUtE	0.104	0.730	0.017	0.010	0.554	0.000
KUtE	0.232	0.000	0.009	0.000	0.005	0.003
CaUtE	0.269	0.000	0.072	0.000	0.001	0.010
MgUtE	0.258	0.483	0.037	0.133	0.358	0.021
NHI	0.068	0.000	0.372	0.807	0.339	0.745
PHI	0.159	0.762	0.302	0.081	0.101	0.000
KHI	0.335	0.000	0.001	0.001	0.092	0.035
CaHI	0.076	0.001	0.117	0.039	0.886	0.638
MgHI	0.245	0.107	0.317	0.464	0.689	0.027

K	Ca	Mg	GF	NDF	ADF	ADL	
0.012	0.084	0.773	0.159	0.071	0.032	0.000	
0.684	0.082	0.001	0.001	0.000	0.001	0.137	
0.011	0.436	0.000	0.005	0.006	0.035	0.411	
0.139	0.263	0.000	0.000	0.000	0.000	0.474	
0.909	0.641	0.124	0.013	0.118	0.117	0.382	
0.184	0.568	0.000	0.000	0.000	0.000	0.561	
0.000	0.639	0.019	0.836	0.607	0.878	0.153	
0.639	0.000	0.019	0.106	0.000	0.000	0.006	
0.019	0.019	0.000	0.000	0.000	0.000	0.476	
0.836	0.106	0.000	0.000	0.000	0.000	0.313	
0.607	0.000	0.000	0.000	0.000	0.000	0.035	
0.878	0.000	0.000	0.000	0.000	0.000	0.008	
0.153	0.006	0.476	0.313	0.035	0.008	0.000	
0.191	0.062	0.000	0.016	0.000	0.005	0.910	
0.712	0.000	0.000	0.000	0.000	0.000	0.050	
0.764	0.000	0.000	0.000	0.000	0.000	0.008	
0.002	0.487	0.101	0.844	0.758	0.932	0.003	
0.006	0.712	0.016	0.281	0.347	0.628	0.001	
0.118	0.078	0.451	0.125	0.040	0.017	0.000	
0.009	0.843	0.494	0.437	0.630	0.477	0.002	
0.000	0.925	0.003	0.234	0.206	0.382	0.007	
0.491	0.267	0.378	0.221	0.295	0.317	0.174	
0.131	0.234	0.000	0.004	0.004	0.005	0.148	
0.184	0.180	0.000	0.002	0.001	0.001	0.198	
0.824	0.564	0.039	0.009	0.078	0.099	0.580	
0.014	0.203	0.001	0.061	0.017	0.023	0.252	
0.345	0.287	0.936	0.683	0.527	0.630	0.154	
0.297	0.392	0.010	0.002	0.068	0.057	0.286	
0.040	0.056	0.000	0.049	0.001	0.005	0.317	
0.913	0.573	0.138	0.401	0.301	0.337	0.533	
0.014	0.628	0.024	0.113	0.172	0.203	0.321	

Hemicellulose	Cellulose	RFV	NU	PU	KU	CaU
0.743	0.095	0.017	0.000	0.000	0.000	0.000
0.008	0.001	0.000	0.419	0.327	0.085	0.312
0.001	0.016	0.016	0.045	0.002	0.508	0.175
0.007	0.000	0.000	0.045	0.054	0.300	0.914
0.326	0.125	0.203	0.169	0.173	0.986	0.856
0.013	0.000	0.000	0.009	0.000	0.513	0.223
0.191	0.712	0.764	0.002	0.006	0.118	0.009
0.062	0.000	0.000	0.487	0.712	0.078	0.843
0.000	0.000	0.000	0.101	0.016	0.451	0.494
0.016	0.000	0.000	0.844	0.281	0.125	0.437
0.000	0.000	0.000	0.758	0.347	0.040	0.630
0.005	0.000	0.000	0.932	0.628	0.017	0.477
0.910	0.050	0.008	0.003	0.001	0.000	0.002
0.000	0.002	0.000	0.207	0.064	0.596	0.766
0.002	0.000	0.000	0.747	0.334	0.056	0.739
0.000	0.000	0.000	0.802	0.679	0.007	0.297
0.207	0.747	0.802	0.000	0.000	0.000	0.000
0.064	0.334	0.679	0.000	0.000	0.000	0.000
0.596	0.056	0.007	0.000	0.000	0.000	0.000
0.766	0.739	0.297	0.000	0.000	0.000	0.000
0.063	0.185	0.460	0.000	0.000	0.000	0.000
0.432	0.387	0.183	0.516	0.783	0.213	0.223
0.045	0.001	0.015	0.012	0.000	0.157	0.018
0.017	0.001	0.000	0.240	0.212	0.135	0.478
0.164	0.095	0.046	0.391	0.359	0.249	0.146
0.078	0.008	0.044	0.185	0.045	0.487	0.066
0.421	0.764	0.406	0.399	0.403	0.107	0.096
0.346	0.026	0.137	0.091	0.019	0.248	0.236
0.004	0.004	0.001	0.306	0.193	0.193	0.772
0.392	0.354	0.079	0.565	0.873	0.068	0.119
0.267	0.131	0.305	0.380	0.225	0.511	0.273

MgU	NUtE	PUtE	KUtE	CaUtE	MgUtE	NHI
0.000	0.150	0.104	0.232	0.269	0.258	0.068
0.463	0.000	0.730	0.000	0.000	0.483	0.000
0.001	0.121	0.017	0.009	0.072	0.037	0.372
0.050	0.728	0.010	0.000	0.000	0.133	0.807
0.315	0.225	0.554	0.005	0.001	0.358	0.339
0.001	0.659	0.000	0.003	0.010	0.021	0.745
0.000	0.491	0.131	0.184	0.824	0.014	0.345
0.925	0.267	0.234	0.180	0.564	0.203	0.287
0.003	0.378	0.000	0.000	0.039	0.001	0.936
0.234	0.221	0.004	0.002	0.009	0.061	0.683
0.206	0.295	0.004	0.001	0.078	0.017	0.527
0.382	0.317	0.005	0.001	0.099	0.023	0.630
0.007	0.174	0.148	0.198	0.580	0.252	0.154
0.063	0.432	0.045	0.017	0.164	0.078	0.421
0.185	0.387	0.001	0.001	0.095	0.008	0.764
0.460	0.183	0.015	0.000	0.046	0.044	0.406
0.000	0.516	0.012	0.240	0.391	0.185	0.399
0.000	0.783	0.000	0.212	0.359	0.045	0.403
0.000	0.213	0.157	0.135	0.249	0.487	0.107
0.000	0.223	0.018	0.478	0.146	0.066	0.096
0.000	0.546	0.000	0.254	0.726	0.005	0.193
0.546	0.000	0.075	0.001	0.000	0.013	0.000
0.000	0.075	0.000	0.547	0.783	0.000	0.007
0.254	0.001	0.547	0.000	0.000	0.838	0.001
0.726	0.000	0.783	0.000	0.000	0.131	0.001
0.005	0.013	0.000	0.838	0.131	0.000	0.001
0.193	0.000	0.007	0.001	0.001	0.001	0.000
0.016	0.070	0.000	0.800	0.736	0.005	0.009
0.179	0.001	0.440	0.000	0.000	0.678	0.002
0.771	0.026	0.922	0.004	0.005	0.624	0.032
0.059	0.003	0.002	0.417	0.158	0.000	0.000

PHI	KHI	CaHI	MgHI
0.159	0.335	0.076	0.245
0.762	0.000	0.001	0.107
0.302	0.001	0.117	0.317
0.081	0.001	0.039	0.464
0.101	0.092	0.886	0.689
0.000	0.035	0.638	0.027
0.297	0.040	0.913	0.014
0.392	0.056	0.573	0.628
0.010	0.000	0.138	0.024
0.002	0.049	0.401	0.113
0.068	0.001	0.301	0.172
0.057	0.005	0.337	0.203
0.286	0.317	0.533	0.321
0.346	0.004	0.392	0.267
0.026	0.004	0.354	0.131
0.137	0.001	0.079	0.305
0.091	0.306	0.565	0.380
0.019	0.193	0.873	0.225
0.248	0.193	0.068	0.511
0.236	0.772	0.119	0.273
0.016	0.179	0.771	0.059
0.070	0.001	0.026	0.003
0.000	0.440	0.922	0.002
0.800	0.000	0.004	0.417
0.736	0.000	0.005	0.158
0.005	0.678	0.624	0.000
0.009	0.002	0.032	0.000
0.000	0.578	0.070	0.000
0.578	0.000	0.002	0.420
0.070	0.002	0.000	0.027
0.000	0.420	0.027	0.000

	Straw yield	HI	Ash	CP	CF	P
Straw yield	1.000	-0.314	-0.561	0.570	0.031	0.436
HI	-0.314	1.000	-0.085	0.030	0.135	0.069
Ash	-0.561	-0.085	1.000	-0.223	-0.396	-0.354
CP	0.570	0.030	-0.223	1.000	-0.182	0.240
CF	0.031	0.135	-0.396	-0.182	1.000	0.001
P	0.436	0.069	-0.354	0.240	0.001	1.000
K	-0.369	0.006	0.674	-0.423	0.059	-0.208
Ca	-0.627	-0.012	0.561	-0.564	0.020	-0.429
Mg	0.592	0.123	-0.567	0.458	0.051	0.646
GF	-0.409	-0.203	0.213	-0.373	-0.173	-0.283
NDF	-0.357	-0.095	0.260	-0.184	-0.386	-0.274
ADF	-0.399	-0.087	0.234	-0.284	-0.379	-0.324
ADL	-0.053	-0.115	-0.156	0.098	-0.338	0.035
Hemicellulose	-0.125	-0.082	0.230	0.132	-0.261	-0.051
Cellulose	-0.420	-0.074	0.280	-0.326	-0.346	-0.357
RFV	0.379	0.088	-0.250	0.210	0.374	0.335
NU	0.811	0.144	-0.478	0.858	-0.005	0.379
PU	0.709	0.272	-0.571	0.476	0.126	0.811
KU	0.892	-0.148	-0.344	0.427	0.065	0.473
CaU	0.611	-0.303	-0.210	0.163	0.071	0.236
MgU	0.835	0.008	-0.642	0.560	0.090	0.643
NUtE	-0.492	0.743	-0.030	-0.582	0.211	-0.027
PUtE	-0.606	0.444	0.324	-0.284	0.127	-0.807
KUtE	-0.133	0.875	-0.378	0.230	0.101	0.086
CaUtE	0.114	0.814	-0.370	0.362	0.047	0.286
MgUtE	-0.802	0.508	0.474	-0.435	0.096	-0.581
NHI	-0.757	0.625	0.261	-0.719	0.188	-0.250
PHI	-0.507	0.632	0.203	-0.143	0.196	-0.688
KHI	-0.083	0.868	-0.341	0.168	0.070	0.337
CaHI	0.056	0.618	-0.289	0.485	-0.005	0.167
MgHI	-0.627	0.656	0.309	-0.273	0.080	-0.353

K	Ca	Mg	GF	NDF	ADF	ADL
-0.369	-0.627	0.592	-0.409	-0.357	-0.399	-0.053
0.006	-0.012	0.123	-0.203	-0.095	-0.087	-0.115
0.674	0.561	-0.567	0.213	0.260	0.234	-0.156
-0.423	-0.564	0.458	-0.373	-0.184	-0.284	0.098
0.059	0.020	0.051	-0.173	-0.386	-0.379	-0.338
-0.208	-0.429	0.646	-0.283	-0.274	-0.324	0.035
1.000	0.503	-0.521	0.022	-0.138	-0.109	-0.594
0.503	1.000	-0.430	0.029	-0.105	-0.029	-0.314
-0.521	-0.430	1.000	-0.603	-0.513	-0.566	0.197
0.022	0.029	-0.603	1.000	0.864	0.881	0.347
-0.138	-0.105	-0.513	0.864	1.000	0.961	0.582
-0.109	-0.029	-0.566	0.881	0.961	1.000	0.514
-0.594	-0.314	0.197	0.347	0.582	0.514	1.000
-0.158	-0.254	-0.194	0.505	0.731	0.515	0.537
-0.008	0.025	-0.648	0.890	0.933	0.987	0.371
0.131	0.084	0.533	-0.867	-0.990	-0.978	-0.578
-0.405	-0.651	0.621	-0.512	-0.366	-0.428	-0.071
-0.276	-0.624	0.723	-0.501	-0.449	-0.488	-0.131
0.033	-0.483	0.458	-0.462	-0.469	-0.495	-0.360
-0.036	0.204	0.431	-0.521	-0.595	-0.572	-0.375
-0.469	-0.582	0.924	-0.612	-0.537	-0.582	0.053
0.197	0.222	-0.133	0.056	0.034	0.091	-0.103
0.229	0.537	-0.496	0.071	0.105	0.158	-0.106
-0.461	-0.241	0.298	-0.145	0.029	0.018	0.199
-0.262	-0.559	0.299	-0.180	-0.008	-0.023	0.079
0.470	0.476	-0.745	0.381	0.330	0.377	-0.192
0.391	0.518	-0.339	0.197	0.130	0.203	-0.155
0.234	0.274	-0.404	0.010	0.041	0.076	-0.222
-0.360	-0.259	0.338	-0.141	-0.015	-0.013	0.051
-0.334	-0.491	0.232	-0.152	0.010	-0.019	0.105
0.400	0.202	-0.616	0.353	0.326	0.366	-0.247

	Straw yield	HI	Ash	CP	CF	P
Straw yield	1.000	-0.317	-0.194	-0.134	0.019	0.177
HI	-0.317	1.000	-0.482	0.729	-0.507	0.507
Ash	-0.194	-0.482	1.000	-0.395	0.188	-0.518
CP	-0.134	0.729	-0.395	1.000	-0.503	0.712
CF	0.019	-0.507	0.188	-0.503	1.000	-0.514
P	0.177	0.507	-0.518	0.712	-0.514	1.000
K	-0.475	-0.082	0.483	-0.292	-0.023	-0.264
Ca	-0.339	0.341	-0.156	0.223	0.094	0.115
Mg	-0.058	0.621	-0.706	0.767	-0.303	0.807
GF	0.279	-0.584	0.527	-0.644	0.470	-0.793
NDF	0.353	-0.641	0.514	-0.712	0.308	-0.673
ADF	0.413	-0.609	0.407	-0.692	0.309	-0.658
ADL	0.739	-0.294	-0.165	-0.144	0.175	0.117
Hemicellulose	0.066	-0.497	0.613	-0.508	0.196	-0.472
Cellulose	0.328	-0.604	0.459	-0.716	0.302	-0.718
RFV	-0.456	0.663	-0.461	0.704	-0.253	0.625
NU	0.834	0.162	-0.389	0.389	-0.273	0.493
PU	0.804	0.196	-0.559	0.375	-0.270	0.645
KU	0.981	-0.338	-0.133	-0.207	0.003	0.132
CaU	0.922	-0.202	-0.269	-0.022	0.037	0.243
MgU	0.786	0.147	-0.609	0.381	-0.201	0.613
NUtE	-0.285	0.713	-0.306	0.070	-0.241	0.089
PUtE	-0.320	-0.070	0.457	-0.488	0.119	-0.710
KUtE	-0.238	0.963	-0.491	0.777	-0.523	0.553
CaUtE	-0.221	0.874	-0.351	0.640	-0.608	0.487
MgUtE	-0.225	0.141	0.403	-0.297	-0.184	-0.443
NHI	-0.356	0.686	-0.179	0.049	-0.191	-0.066
PHI	-0.279	0.061	0.206	-0.342	0.322	-0.777
KHI	-0.193	0.850	-0.610	0.615	-0.330	0.408
CaHI	-0.347	0.581	-0.309	0.400	-0.029	0.095
MgHI	-0.232	0.317	0.200	-0.147	-0.081	-0.424

K	Ca	Mg	GF	NDF	ADF	ADL
-0.475	-0.339	-0.058	0.279	0.353	0.413	0.739
-0.082	0.341	0.621	-0.584	-0.641	-0.609	-0.294
0.483	-0.156	-0.706	0.527	0.514	0.407	-0.165
-0.292	0.223	0.767	-0.644	-0.712	-0.692	-0.144
-0.023	0.094	-0.303	0.470	0.308	0.309	0.175
-0.264	0.115	0.807	-0.793	-0.673	-0.658	0.117
1.000	0.095	-0.450	-0.042	0.104	0.031	-0.282
0.095	1.000	0.447	-0.318	-0.670	-0.694	-0.513
-0.450	0.447	1.000	-0.788	-0.883	-0.847	-0.143
-0.042	-0.318	-0.788	1.000	0.808	0.830	0.202
0.104	-0.670	-0.883	0.808	1.000	0.966	0.407
0.031	-0.694	-0.847	0.830	0.966	1.000	0.497
-0.282	-0.513	-0.143	0.202	0.407	0.497	1.000
0.260	-0.364	-0.662	0.457	0.730	0.529	0.023
0.074	-0.664	-0.881	0.854	0.969	0.992	0.380
-0.061	0.653	0.856	-0.794	-0.968	-0.955	-0.502
-0.566	-0.140	0.323	-0.040	-0.062	0.017	0.558
-0.513	-0.074	0.458	-0.215	-0.188	-0.098	0.591
-0.308	-0.345	-0.151	0.302	0.398	0.454	0.735
-0.494	0.040	0.138	0.156	0.097	0.143	0.560
-0.651	0.019	0.552	-0.237	-0.251	-0.175	0.506
0.138	0.221	0.177	-0.243	-0.209	-0.200	-0.270
0.298	-0.237	-0.677	0.541	0.541	0.525	-0.286
-0.264	0.266	0.671	-0.570	-0.617	-0.594	-0.256
-0.045	-0.116	0.400	-0.491	-0.345	-0.324	-0.111
0.465	-0.253	-0.607	0.366	0.456	0.436	-0.229
0.189	0.213	0.016	-0.082	-0.127	-0.097	-0.282
0.208	0.171	-0.486	0.578	0.356	0.371	-0.213
-0.397	0.371	0.661	-0.382	-0.589	-0.528	-0.200
-0.022	0.113	0.293	-0.169	-0.207	-0.192	-0.125
0.467	0.098	-0.433	0.312	0.271	0.253	-0.199

Hemicellulose	Cellulose	RFV	NU	PU	KU	CaU	
0.066	0.328	-0.456	0.834	0.804	0.981	0.922	
-0.497	-0.604	0.663	0.162	0.196	-0.338	-0.202	
0.613	0.459	-0.461	-0.389	-0.559	-0.133	-0.269	
-0.508	-0.716	0.704	0.389	0.375	-0.207	-0.022	
0.196	0.302	-0.253	-0.273	-0.270	0.003	0.037	
-0.472	-0.718	0.625	0.493	0.645	0.132	0.243	
0.260	0.074	-0.061	-0.566	-0.513	-0.308	-0.494	
-0.364	-0.664	0.653	-0.140	-0.074	-0.345	0.040	
-0.662	-0.881	0.856	0.323	0.458	-0.151	0.138	
0.457	0.854	-0.794	-0.040	-0.215	0.302	0.156	
0.730	0.969	-0.968	-0.062	-0.188	0.398	0.097	
0.529	0.992	-0.955	0.017	-0.098	0.454	0.143	
0.023	0.380	-0.502	0.558	0.591	0.735	0.560	
1.000	0.560	-0.653	-0.251	-0.361	0.107	-0.060	
0.560	1.000	-0.943	-0.065	-0.193	0.372	0.067	
-0.653	-0.943	1.000	-0.051	0.084	-0.503	-0.208	
-0.251	-0.065	-0.051	1.000	0.934	0.791	0.840	
-0.361	-0.193	0.084	0.934	1.000	0.772	0.824	
0.107	0.372	-0.503	0.791	0.772	1.000	0.891	
-0.060	0.067	-0.208	0.840	0.824	0.891	1.000	
-0.362	-0.263	0.148	0.909	0.957	0.718	0.858	
-0.158	-0.173	0.264	-0.131	-0.056	-0.247	-0.242	
0.389	0.603	-0.462	-0.475	-0.631	-0.280	-0.453	
-0.456	-0.594	0.639	0.234	0.248	-0.295	-0.143	
-0.276	-0.328	0.387	0.172	0.184	-0.230	-0.287	
0.345	0.499	-0.391	-0.263	-0.389	-0.140	-0.359	
-0.161	-0.061	0.167	-0.169	-0.168	-0.317	-0.327	
0.189	0.427	-0.294	-0.331	-0.449	-0.230	-0.236	
-0.538	-0.532	0.590	0.205	0.259	-0.259	-0.059	
-0.172	-0.186	0.344	-0.116	-0.032	-0.356	-0.307	
0.221	0.298	-0.205	-0.176	-0.242	-0.132	-0.219	

MgU	NUtE	PUtE	KUtE	CaUtE	MgUtE	NHI
0.786	-0.285	-0.320	-0.238	-0.221	-0.225	-0.356
0.147	0.713	-0.070	0.963	0.874	0.141	0.686
-0.609	-0.306	0.457	-0.491	-0.351	0.403	-0.179
0.381	0.070	-0.488	0.777	0.640	-0.297	0.049
-0.201	-0.241	0.119	-0.523	-0.608	-0.184	-0.191
0.613	0.089	-0.710	0.553	0.487	-0.443	-0.066
-0.651	0.138	0.298	-0.264	-0.045	0.465	0.189
0.019	0.221	-0.237	0.266	-0.116	-0.253	0.213
0.552	0.177	-0.677	0.671	0.400	-0.607	0.016
-0.237	-0.243	0.541	-0.570	-0.491	0.366	-0.082
-0.251	-0.209	0.541	-0.617	-0.345	0.456	-0.127
-0.175	-0.200	0.525	-0.594	-0.324	0.436	-0.097
0.506	-0.270	-0.286	-0.256	-0.111	-0.229	-0.282
-0.362	-0.158	0.389	-0.456	-0.276	0.345	-0.161
-0.263	-0.173	0.603	-0.594	-0.328	0.499	-0.061
0.148	0.264	-0.462	0.639	0.387	-0.391	0.167
0.909	-0.131	-0.475	0.234	0.172	-0.263	-0.169
0.957	-0.056	-0.631	0.248	0.184	-0.389	-0.168
0.718	-0.247	-0.280	-0.295	-0.230	-0.140	-0.317
0.858	-0.242	-0.453	-0.143	-0.287	-0.359	-0.327
1.000	-0.121	-0.669	0.227	0.071	-0.520	-0.258
-0.121	1.000	0.348	0.623	0.639	0.473	0.879
-0.669	0.348	1.000	-0.121	0.055	0.868	0.507
0.227	0.623	-0.121	1.000	0.887	0.041	0.583
0.071	0.639	0.055	0.887	1.000	0.298	0.617
-0.520	0.473	0.868	0.041	0.298	1.000	0.604
-0.258	0.879	0.507	0.583	0.617	0.604	1.000
-0.460	0.354	0.630	-0.051	-0.068	0.522	0.494
0.267	0.593	-0.155	0.885	0.682	-0.084	0.566
-0.059	0.428	0.020	0.537	0.521	0.099	0.414
-0.368	0.545	0.579	0.163	0.280	0.770	0.665

PHI	KHI	CaHI	MgHI
-0.279	-0.193	-0.347	-0.232
0.061	0.850	0.581	0.317
0.206	-0.610	-0.309	0.200
-0.342	0.615	0.400	-0.147
0.322	-0.330	-0.029	-0.081
-0.777	0.408	0.095	-0.424
0.208	-0.397	-0.022	0.467
0.171	0.371	0.113	0.098
-0.486	0.661	0.293	-0.433
0.578	-0.382	-0.169	0.312
0.356	-0.589	-0.207	0.271
0.371	-0.528	-0.192	0.253
-0.213	-0.200	-0.125	-0.199
0.189	-0.538	-0.172	0.221
0.427	-0.532	-0.186	0.298
-0.294	0.590	0.344	-0.205
-0.331	0.205	-0.116	-0.176
-0.449	0.259	-0.032	-0.242
-0.230	-0.259	-0.356	-0.132
-0.236	-0.059	-0.307	-0.219
-0.460	0.267	-0.059	-0.368
0.354	0.593	0.428	0.545
0.630	-0.155	0.020	0.579
-0.051	0.885	0.537	0.163
-0.068	0.682	0.521	0.280
0.522	-0.084	0.099	0.770
0.494	0.566	0.414	0.665
1.000	0.112	0.355	0.768
0.112	1.000	0.575	0.162
0.355	0.575	1.000	0.427
0.768	0.162	0.427	1.000

	Straw yield	HI	Ash	CP	CF	P
Straw yield	0.000	0.107	0.333	0.506	0.923	0.378
HI	0.107	0.000	0.011	0.000	0.007	0.007
Ash	0.333	0.011	0.000	0.042	0.348	0.006
CP	0.506	0.000	0.042	0.000	0.007	0.000
CF	0.923	0.007	0.348	0.007	0.000	0.006
P	0.378	0.007	0.006	0.000	0.006	0.000
K	0.012	0.684	0.011	0.139	0.909	0.184
Ca	0.084	0.082	0.436	0.263	0.641	0.568
Mg	0.773	0.001	0.000	0.000	0.124	0.000
GF	0.159	0.001	0.005	0.000	0.013	0.000
NDF	0.071	0.000	0.006	0.000	0.118	0.000
ADF	0.032	0.001	0.035	0.000	0.117	0.000
ADL	0.000	0.137	0.411	0.474	0.382	0.561
Hemicellulose	0.743	0.008	0.001	0.007	0.326	0.013
Cellulose	0.095	0.001	0.016	0.000	0.125	0.000
RFV	0.017	0.000	0.016	0.000	0.203	0.000
NU	0.000	0.419	0.045	0.045	0.169	0.009
PU	0.000	0.327	0.002	0.054	0.173	0.000
KU	0.000	0.085	0.508	0.300	0.986	0.513
CaU	0.000	0.312	0.175	0.914	0.856	0.223
MgU	0.000	0.463	0.001	0.050	0.315	0.001
NUtE	0.150	0.000	0.121	0.728	0.225	0.659
PUtE	0.104	0.730	0.017	0.010	0.554	0.000
KUtE	0.232	0.000	0.009	0.000	0.005	0.003
CaUtE	0.269	0.000	0.072	0.000	0.001	0.010
MgUtE	0.258	0.483	0.037	0.133	0.358	0.021
NHI	0.068	0.000	0.372	0.807	0.339	0.745
PHI	0.159	0.762	0.302	0.081	0.101	0.000
KHI	0.335	0.000	0.001	0.001	0.092	0.035
CaHI	0.076	0.001	0.117	0.039	0.886	0.638
MgHI	0.245	0.107	0.317	0.464	0.689	0.027

K	Ca	Mg	GF	NDF	ADF	ADL
0.012	0.084	0.773	0.159	0.071	0.032	0.000
0.684	0.082	0.001	0.001	0.000	0.001	0.137
0.011	0.436	0.000	0.005	0.006	0.035	0.411
0.139	0.263	0.000	0.000	0.000	0.000	0.474
0.909	0.641	0.124	0.013	0.118	0.117	0.382
0.184	0.568	0.000	0.000	0.000	0.000	0.561
0.000	0.639	0.019	0.836	0.607	0.878	0.153
0.639	0.000	0.019	0.106	0.000	0.000	0.006
0.019	0.019	0.000	0.000	0.000	0.000	0.476
0.836	0.106	0.000	0.000	0.000	0.000	0.313
0.607	0.000	0.000	0.000	0.000	0.000	0.035
0.878	0.000	0.000	0.000	0.000	0.000	0.008
0.153	0.006	0.476	0.313	0.035	0.008	0.000
0.191	0.062	0.000	0.016	0.000	0.005	0.910
0.712	0.000	0.000	0.000	0.000	0.000	0.050
0.764	0.000	0.000	0.000	0.000	0.000	0.008
0.002	0.487	0.101	0.844	0.758	0.932	0.003
0.006	0.712	0.016	0.281	0.347	0.628	0.001
0.118	0.078	0.451	0.125	0.040	0.017	0.000
0.009	0.843	0.494	0.437	0.630	0.477	0.002
0.000	0.925	0.003	0.234	0.206	0.382	0.007
0.491	0.267	0.378	0.221	0.295	0.317	0.174
0.131	0.234	0.000	0.004	0.004	0.005	0.148
0.184	0.180	0.000	0.002	0.001	0.001	0.198
0.824	0.564	0.039	0.009	0.078	0.099	0.580
0.014	0.203	0.001	0.061	0.017	0.023	0.252
0.345	0.287	0.936	0.683	0.527	0.630	0.154
0.297	0.392	0.010	0.002	0.068	0.057	0.286
0.040	0.056	0.000	0.049	0.001	0.005	0.317
0.913	0.573	0.138	0.401	0.301	0.337	0.533
0.014	0.628	0.024	0.113	0.172	0.203	0.321

Hemicellulose	Cellulose	RFV	NU	PU	KU	CaU	
0.743	0.095	0.017	0.000	0.000	0.000	0.000
0.008	0.001	0.000	0.419	0.327	0.085	0.312
0.001	0.016	0.016	0.045	0.002	0.508	0.175
0.007	0.000	0.000	0.045	0.054	0.300	0.914
0.326	0.125	0.203	0.169	0.173	0.986	0.856
0.013	0.000	0.000	0.009	0.000	0.513	0.223
0.191	0.712	0.764	0.002	0.006	0.118	0.009
0.062	0.000	0.000	0.487	0.712	0.078	0.843
0.000	0.000	0.000	0.101	0.016	0.451	0.494
0.016	0.000	0.000	0.844	0.281	0.125	0.437
0.000	0.000	0.000	0.758	0.347	0.040	0.630
0.005	0.000	0.000	0.932	0.628	0.017	0.477
0.910	0.050	0.008	0.003	0.001	0.000	0.002
0.000	0.002	0.000	0.207	0.064	0.596	0.766
0.002	0.000	0.000	0.747	0.334	0.056	0.739
0.000	0.000	0.000	0.802	0.679	0.007	0.297
0.207	0.747	0.802	0.000	0.000	0.000	0.000
0.064	0.334	0.679	0.000	0.000	0.000	0.000
0.596	0.056	0.007	0.000	0.000	0.000	0.000
0.766	0.739	0.297	0.000	0.000	0.000	0.000
0.063	0.185	0.460	0.000	0.000	0.000	0.000
0.432	0.387	0.183	0.516	0.783	0.213	0.223
0.045	0.001	0.015	0.012	0.000	0.157	0.018
0.017	0.001	0.000	0.240	0.212	0.135	0.478
0.164	0.095	0.046	0.391	0.359	0.249	0.146
0.078	0.008	0.044	0.185	0.045	0.487	0.066
0.421	0.764	0.406	0.399	0.403	0.107	0.096
0.346	0.026	0.137	0.091	0.019	0.248	0.236
0.004	0.004	0.001	0.306	0.193	0.193	0.772
0.392	0.354	0.079	0.565	0.873	0.068	0.119
0.267	0.131	0.305	0.380	0.225	0.511	0.273

MgU	NUtE	PUtE	KUtE	CaUtE	MgUtE	NHI
0.000	0.150	0.104	0.232	0.269	0.258	0.068
0.463	0.000	0.730	0.000	0.000	0.483	0.000
0.001	0.121	0.017	0.009	0.072	0.037	0.372
0.050	0.728	0.010	0.000	0.000	0.133	0.807
0.315	0.225	0.554	0.005	0.001	0.358	0.339
0.001	0.659	0.000	0.003	0.010	0.021	0.745
0.000	0.491	0.131	0.184	0.824	0.014	0.345
0.925	0.267	0.234	0.180	0.564	0.203	0.287
0.003	0.378	0.000	0.000	0.039	0.001	0.936
0.234	0.221	0.004	0.002	0.009	0.061	0.683
0.206	0.295	0.004	0.001	0.078	0.017	0.527
0.382	0.317	0.005	0.001	0.099	0.023	0.630
0.007	0.174	0.148	0.198	0.580	0.252	0.154
0.063	0.432	0.045	0.017	0.164	0.078	0.421
0.185	0.387	0.001	0.001	0.095	0.008	0.764
0.460	0.183	0.015	0.000	0.046	0.044	0.406
0.000	0.516	0.012	0.240	0.391	0.185	0.399
0.000	0.783	0.000	0.212	0.359	0.045	0.403
0.000	0.213	0.157	0.135	0.249	0.487	0.107
0.000	0.223	0.018	0.478	0.146	0.066	0.096
0.000	0.546	0.000	0.254	0.726	0.005	0.193
0.546	0.000	0.075	0.001	0.000	0.013	0.000
0.000	0.075	0.000	0.547	0.783	0.000	0.007
0.254	0.001	0.547	0.000	0.000	0.838	0.001
0.726	0.000	0.783	0.000	0.000	0.131	0.001
0.005	0.013	0.000	0.838	0.131	0.000	0.001
0.193	0.000	0.007	0.001	0.001	0.001	0.000
0.016	0.070	0.000	0.800	0.736	0.005	0.009
0.179	0.001	0.440	0.000	0.000	0.678	0.002
0.771	0.026	0.922	0.004	0.005	0.624	0.032
0.059	0.003	0.002	0.417	0.158	0.000	0.000

PHI	KHI	CaHI	MgHI
0.159	0.335	0.076	0.245
0.762	0.000	0.001	0.107
0.302	0.001	0.117	0.317
0.081	0.001	0.039	0.464
0.101	0.092	0.886	0.689
0.000	0.035	0.638	0.027
0.297	0.040	0.913	0.014
0.392	0.056	0.573	0.628
0.010	0.000	0.138	0.024
0.002	0.049	0.401	0.113
0.068	0.001	0.301	0.172
0.057	0.005	0.337	0.203
0.286	0.317	0.533	0.321
0.346	0.004	0.392	0.267
0.026	0.004	0.354	0.131
0.137	0.001	0.079	0.305
0.091	0.306	0.565	0.380
0.019	0.193	0.873	0.225
0.248	0.193	0.068	0.511
0.236	0.772	0.119	0.273
0.016	0.179	0.771	0.059
0.070	0.001	0.026	0.003
0.000	0.440	0.922	0.002
0.800	0.000	0.004	0.417
0.736	0.000	0.005	0.158
0.005	0.678	0.624	0.000
0.009	0.002	0.032	0.000
0.000	0.578	0.070	0.000
0.578	0.000	0.002	0.420
0.070	0.002	0.000	0.027
0.000	0.420	0.027	0.000

	Straw yield	HI	Ash	CP	CF	P
Straw yield	1.000	-0.314	-0.561	0.570	0.031	0.436
HI	-0.314	1.000	-0.085	0.030	0.135	0.069
Ash	-0.561	-0.085	1.000	-0.223	-0.396	-0.354
CP	0.570	0.030	-0.223	1.000	-0.182	0.240
CF	0.031	0.135	-0.396	-0.182	1.000	0.001
P	0.436	0.069	-0.354	0.240	0.001	1.000
K	-0.369	0.006	0.674	-0.423	0.059	-0.208
Ca	-0.627	-0.012	0.561	-0.564	0.020	-0.429
Mg	0.592	0.123	-0.567	0.458	0.051	0.646
GF	-0.409	-0.203	0.213	-0.373	-0.173	-0.283
NDF	-0.357	-0.095	0.260	-0.184	-0.386	-0.274
ADF	-0.399	-0.087	0.234	-0.284	-0.379	-0.324
ADL	-0.053	-0.115	-0.156	0.098	-0.338	0.035
Hemicellulose	-0.125	-0.082	0.230	0.132	-0.261	-0.051
Cellulose	-0.420	-0.074	0.280	-0.326	-0.346	-0.357
RFV	0.379	0.088	-0.250	0.210	0.374	0.335
NU	0.811	0.144	-0.478	0.858	-0.005	0.379
PU	0.709	0.272	-0.571	0.476	0.126	0.811
KU	0.892	-0.148	-0.344	0.427	0.065	0.473
CaU	0.611	-0.303	-0.210	0.163	0.071	0.236
MgU	0.835	0.008	-0.642	0.560	0.090	0.643
NUtE	-0.492	0.743	-0.030	-0.582	0.211	-0.027
PUtE	-0.606	0.444	0.324	-0.284	0.127	-0.807
KUtE	-0.133	0.875	-0.378	0.230	0.101	0.086
CaUtE	0.114	0.814	-0.370	0.362	0.047	0.286
MgUtE	-0.802	0.508	0.474	-0.435	0.096	-0.581
NHI	-0.757	0.625	0.261	-0.719	0.188	-0.250
PHI	-0.507	0.632	0.203	-0.143	0.196	-0.688
KHI	-0.083	0.868	-0.341	0.168	0.070	0.337
CaHI	0.056	0.618	-0.289	0.485	-0.005	0.167
MgHI	-0.627	0.656	0.309	-0.273	0.080	-0.353

K	Ca	Mg	GF	NDF	ADF	ADL
-0.369	-0.627	0.592	-0.409	-0.357	-0.399	-0.053
0.006	-0.012	0.123	-0.203	-0.095	-0.087	-0.115
0.674	0.561	-0.567	0.213	0.260	0.234	-0.156
-0.423	-0.564	0.458	-0.373	-0.184	-0.284	0.098
0.059	0.020	0.051	-0.173	-0.386	-0.379	-0.338
-0.208	-0.429	0.646	-0.283	-0.274	-0.324	0.035
1.000	0.503	-0.521	0.022	-0.138	-0.109	-0.594
0.503	1.000	-0.430	0.029	-0.105	-0.029	-0.314
-0.521	-0.430	1.000	-0.603	-0.513	-0.566	0.197
0.022	0.029	-0.603	1.000	0.864	0.881	0.347
-0.138	-0.105	-0.513	0.864	1.000	0.961	0.582
-0.109	-0.029	-0.566	0.881	0.961	1.000	0.514
-0.594	-0.314	0.197	0.347	0.582	0.514	1.000
-0.158	-0.254	-0.194	0.505	0.731	0.515	0.537
-0.008	0.025	-0.648	0.890	0.933	0.987	0.371
0.131	0.084	0.533	-0.867	-0.990	-0.978	-0.578
-0.405	-0.651	0.621	-0.512	-0.366	-0.428	-0.071
-0.276	-0.624	0.723	-0.501	-0.449	-0.488	-0.131
0.033	-0.483	0.458	-0.462	-0.469	-0.495	-0.360
-0.036	0.204	0.431	-0.521	-0.595	-0.572	-0.375
-0.469	-0.582	0.924	-0.612	-0.537	-0.582	0.053
0.197	0.222	-0.133	0.056	0.034	0.091	-0.103
0.229	0.537	-0.496	0.071	0.105	0.158	-0.106
-0.461	-0.241	0.298	-0.145	0.029	0.018	0.199
-0.262	-0.559	0.299	-0.180	-0.008	-0.023	0.079
0.470	0.476	-0.745	0.381	0.330	0.377	-0.192
0.391	0.518	-0.339	0.197	0.130	0.203	-0.155
0.234	0.274	-0.404	0.010	0.041	0.076	-0.222
-0.360	-0.259	0.338	-0.141	-0.015	-0.013	0.051
-0.334	-0.491	0.232	-0.152	0.010	-0.019	0.105
0.400	0.202	-0.616	0.353	0.326	0.366	-0.247

Hemicellulose	Cellulose	RFV	NU	PU	KU	CaU
-0.125	-0.420	0.379	0.811	0.709	0.892	0.611
-0.082	-0.074	0.088	0.144	0.272	-0.148	-0.303
0.230	0.280	-0.250	-0.478	-0.571	-0.344	-0.210
0.132	-0.326	0.210	0.858	0.476	0.427	0.163
-0.261	-0.346	0.374	-0.005	0.126	0.065	0.071
-0.051	-0.357	0.335	0.379	0.811	0.473	0.236
-0.158	-0.008	0.131	-0.405	-0.276	0.033	-0.036
-0.254	0.025	0.084	-0.651	-0.624	-0.483	0.204
-0.194	-0.648	0.533	0.621	0.723	0.458	0.431
0.505	0.890	-0.867	-0.512	-0.501	-0.462	-0.521
0.731	0.933	-0.990	-0.366	-0.449	-0.469	-0.595
0.515	0.987	-0.978	-0.428	-0.488	-0.495	-0.572
0.537	0.371	-0.578	-0.071	-0.131	-0.360	-0.375
1.000	0.458	-0.659	-0.077	-0.188	-0.232	-0.437
0.458	1.000	-0.951	-0.449	-0.502	-0.467	-0.548
-0.659	-0.951	1.000	0.388	0.489	0.492	0.609
-0.077	-0.449	0.388	1.000	0.760	0.739	0.395
-0.188	-0.502	0.489	0.760	1.000	0.761	0.360
-0.232	-0.467	0.492	0.739	0.761	1.000	0.617
-0.437	-0.548	0.609	0.395	0.360	0.617	1.000
-0.231	-0.639	0.561	0.800	0.847	0.727	0.539
-0.121	0.117	-0.052	-0.369	0.022	-0.280	-0.323
-0.065	0.189	-0.156	-0.342	-0.603	-0.535	-0.276
0.043	-0.017	-0.032	0.300	0.311	-0.185	-0.287
0.032	-0.039	0.006	0.502	0.577	0.181	-0.340
0.094	0.442	-0.355	-0.532	-0.548	-0.602	-0.569
-0.100	0.248	-0.155	-0.605	-0.319	-0.539	-0.377
-0.060	0.122	-0.089	-0.155	-0.364	-0.382	-0.393
-0.016	-0.023	0.019	0.289	0.475	-0.045	-0.218
0.079	-0.042	-0.014	0.425	0.380	0.033	-0.327
0.107	0.441	-0.347	-0.295	-0.243	-0.391	-0.588

Hemicellulose	Cellulose	RFV	NU	PU	KU	CaU
-0.125	-0.420	0.379	0.811	0.709	0.892	0.611
-0.082	-0.074	0.088	0.144	0.272	-0.148	-0.303
0.230	0.280	-0.250	-0.478	-0.571	-0.344	-0.210
0.132	-0.326	0.210	0.858	0.476	0.427	0.163
-0.261	-0.346	0.374	-0.005	0.126	0.065	0.071
-0.051	-0.357	0.335	0.379	0.811	0.473	0.236
-0.158	-0.008	0.131	-0.405	-0.276	0.033	-0.036
-0.254	0.025	0.084	-0.651	-0.624	-0.483	0.204
-0.194	-0.648	0.533	0.621	0.723	0.458	0.431
0.505	0.890	-0.867	-0.512	-0.501	-0.462	-0.521
0.731	0.933	-0.990	-0.366	-0.449	-0.469	-0.595
0.515	0.987	-0.978	-0.428	-0.488	-0.495	-0.572
0.537	0.371	-0.578	-0.071	-0.131	-0.360	-0.375
1.000	0.458	-0.659	-0.077	-0.188	-0.232	-0.437
0.458	1.000	-0.951	-0.449	-0.502	-0.467	-0.548
-0.659	-0.951	1.000	0.388	0.489	0.492	0.609
-0.077	-0.449	0.388	1.000	0.760	0.739	0.395
-0.188	-0.502	0.489	0.760	1.000	0.761	0.360
-0.232	-0.467	0.492	0.739	0.761	1.000	0.617
-0.437	-0.548	0.609	0.395	0.360	0.617	1.000
-0.231	-0.639	0.561	0.800	0.847	0.727	0.539
-0.121	0.117	-0.052	-0.369	0.022	-0.280	-0.323
-0.065	0.189	-0.156	-0.342	-0.603	-0.535	-0.276
0.043	-0.017	-0.032	0.300	0.311	-0.185	-0.287
0.032	-0.039	0.006	0.502	0.577	0.181	-0.340
0.094	0.442	-0.355	-0.532	-0.548	-0.602	-0.569
-0.100	0.248	-0.155	-0.605	-0.319	-0.539	-0.377
-0.060	0.122	-0.089	-0.155	-0.364	-0.382	-0.393
-0.016	-0.023	0.019	0.289	0.475	-0.045	-0.218
0.079	-0.042	-0.014	0.425	0.380	0.033	-0.327
0.107	0.441	-0.347	-0.295	-0.243	-0.391	-0.588

MgU	NUtE	PUtE	KUtE	CaUtE	MgUtE	NHI
0.835	-0.492	-0.606	-0.133	0.114	-0.802	-0.757
0.008	0.743	0.444	0.875	0.814	0.508	0.625
-0.642	-0.030	0.324	-0.378	-0.370	0.474	0.261
0.560	-0.582	-0.284	0.230	0.362	-0.435	-0.719
0.090	0.211	0.127	0.101	0.047	0.096	0.188
0.643	-0.027	-0.807	0.086	0.286	-0.581	-0.250
-0.469	0.197	0.229	-0.461	-0.262	0.470	0.391
-0.582	0.222	0.537	-0.241	-0.559	0.476	0.518
0.924	-0.133	-0.496	0.298	0.299	-0.745	-0.339
-0.612	0.056	0.071	-0.145	-0.180	0.381	0.197
-0.537	0.034	0.105	0.029	-0.008	0.330	0.130
-0.582	0.091	0.158	0.018	-0.023	0.377	0.203
0.053	-0.103	-0.106	0.199	0.079	-0.192	-0.155
-0.231	-0.121	-0.065	0.043	0.032	0.094	-0.100
-0.639	0.117	0.189	-0.017	-0.039	0.442	0.248
0.561	-0.052	-0.156	-0.032	0.006	-0.355	-0.155
0.800	-0.369	-0.342	0.300	0.502	-0.532	-0.605
0.847	0.022	-0.603	0.311	0.577	-0.548	-0.319
0.727	-0.280	-0.535	-0.185	0.181	-0.602	-0.539
0.539	-0.323	-0.276	-0.287	-0.340	-0.569	-0.377
1.000	-0.255	-0.584	0.176	0.313	-0.806	-0.522
-0.255	1.000	0.459	0.553	0.469	0.592	0.858
-0.584	0.459	1.000	0.334	0.068	0.791	0.598
0.176	0.553	0.334	1.000	0.838	0.269	0.367
0.313	0.469	0.068	0.838	1.000	0.156	0.187
-0.806	0.592	0.791	0.269	0.156	1.000	0.748
-0.522	0.858	0.598	0.367	0.187	0.748	1.000
-0.446	0.548	0.897	0.494	0.358	0.796	0.593
0.224	0.604	0.112	0.920	0.855	0.181	0.380
0.209	0.240	0.036	0.724	0.773	0.101	-0.018
-0.621	0.633	0.593	0.421	0.439	0.915	0.691

PHI	KHI	CaHI	MgHI
-0.507	-0.083	0.056	-0.627
0.632	0.868	0.618	0.656
0.203	-0.341	-0.289	0.309
-0.143	0.168	0.485	-0.273
0.196	0.070	-0.005	0.080
-0.688	0.337	0.167	-0.353
0.234	-0.360	-0.334	0.400
0.274	-0.259	-0.491	0.202
-0.404	0.338	0.232	-0.616
0.010	-0.141	-0.152	0.353
0.041	-0.015	0.010	0.326
0.076	-0.013	-0.019	0.366
-0.222	0.051	0.105	-0.247
-0.060	-0.016	0.079	0.107
0.122	-0.023	-0.042	0.441
-0.089	0.019	-0.014	-0.347
-0.155	0.289	0.425	-0.295
-0.364	0.475	0.380	-0.243
-0.382	-0.045	0.033	-0.391
-0.393	-0.218	-0.327	-0.588
-0.446	0.224	0.209	-0.621
0.548	0.604	0.240	0.633
0.897	0.112	0.036	0.593
0.494	0.920	0.724	0.421
0.358	0.855	0.773	0.439
0.796	0.181	0.101	0.915
0.593	0.380	-0.018	0.691
1.000	0.320	0.302	0.742
0.320	1.000	0.705	0.412
0.302	0.705	1.000	0.329
0.742	0.412	0.329	1.000

	Straw yield	HI	Ash	CP	CF	P
Straw yield	0.000	0.111	0.002	0.002	0.877	0.023
HI	0.111	0.000	0.675	0.883	0.501	0.734
Ash	0.002	0.675	0.000	0.264	0.041	0.070
CP	0.002	0.883	0.264	0.000	0.364	0.227
CF	0.877	0.501	0.041	0.364	0.000	0.996
P	0.023	0.734	0.070	0.227	0.996	0.000
K	0.058	0.975	0.000	0.028	0.769	0.298
Ca	0.000	0.952	0.002	0.002	0.922	0.025
Mg	0.001	0.541	0.002	0.016	0.802	0.000
GF	0.034	0.309	0.285	0.055	0.389	0.153
NDF	0.067	0.636	0.191	0.359	0.047	0.166
ADF	0.039	0.666	0.241	0.151	0.051	0.099
ADL	0.791	0.569	0.438	0.628	0.085	0.861
Hemicellulose	0.535	0.686	0.249	0.511	0.189	0.800
Cellulose	0.029	0.715	0.158	0.097	0.077	0.068
RFV	0.051	0.664	0.209	0.294	0.054	0.087
NU	0.000	0.474	0.012	0.000	0.979	0.051
PU	0.000	0.171	0.002	0.012	0.531	0.000
KU	0.000	0.460	0.079	0.026	0.749	0.013
CaU	0.001	0.124	0.294	0.416	0.726	0.237
MgU	0.000	0.967	0.000	0.002	0.656	0.000
NUtE	0.009	0.000	0.882	0.001	0.291	0.894
PUtE	0.001	0.020	0.100	0.151	0.528	0.000
KUtE	0.508	0.000	0.052	0.249	0.614	0.670
CaUtE	0.571	0.000	0.057	0.064	0.815	0.148
MgUtE	0.000	0.007	0.013	0.024	0.632	0.001
NHI	0.000	0.000	0.189	0.000	0.347	0.209
PHI	0.007	0.000	0.309	0.477	0.327	0.000
KHI	0.682	0.000	0.082	0.403	0.729	0.086
CaHI	0.781	0.001	0.144	0.010	0.980	0.406
MgHI	0.000	0.000	0.117	0.168	0.693	0.071

K	Ca	Mg	GF	NDF	ADF	ADL
0.058	0.000	0.001	0.034	0.067	0.039	0.791
0.975	0.952	0.541	0.309	0.636	0.666	0.569
0.000	0.002	0.002	0.285	0.191	0.241	0.438
0.028	0.002	0.016	0.055	0.359	0.151	0.628
0.769	0.922	0.802	0.389	0.047	0.051	0.085
0.298	0.025	0.000	0.153	0.166	0.099	0.861
0.000	0.007	0.005	0.912	0.493	0.589	0.001
0.007	0.000	0.025	0.888	0.604	0.885	0.111
0.005	0.025	0.000	0.001	0.006	0.002	0.324
0.912	0.888	0.001	0.000	0.000	0.000	0.076
0.493	0.604	0.006	0.000	0.000	0.000	0.001
0.589	0.885	0.002	0.000	0.000	0.000	0.006
0.001	0.111	0.324	0.076	0.001	0.006	0.000
0.431	0.202	0.331	0.007	0.000	0.006	0.004
0.968	0.900	0.000	0.000	0.000	0.000	0.057
0.514	0.677	0.004	0.000	0.000	0.000	0.002
0.036	0.000	0.001	0.006	0.061	0.026	0.725
0.164	0.001	0.000	0.008	0.019	0.010	0.515
0.871	0.011	0.016	0.015	0.014	0.009	0.065
0.857	0.308	0.025	0.005	0.001	0.002	0.054
0.014	0.001	0.000	0.001	0.004	0.001	0.792
0.325	0.266	0.510	0.780	0.867	0.651	0.610
0.250	0.004	0.008	0.724	0.603	0.431	0.599
0.015	0.226	0.131	0.470	0.887	0.927	0.321
0.187	0.002	0.129	0.369	0.967	0.908	0.696
0.013	0.012	0.000	0.050	0.093	0.052	0.337
0.044	0.006	0.083	0.326	0.519	0.310	0.439
0.240	0.167	0.037	0.960	0.838	0.705	0.265
0.065	0.192	0.085	0.484	0.940	0.949	0.800
0.088	0.009	0.243	0.448	0.960	0.926	0.601
0.039	0.312	0.001	0.071	0.097	0.060	0.213

Hemicellulose	Cellulose	RFV	NU	PU	KU	CaU	
0.535	0.029	0.051	0.000	0.000	0.000	0.001	0.000
0.686	0.715	0.664	0.474	0.171	0.460	0.124	0.000
0.249	0.158	0.209	0.012	0.002	0.079	0.294	0.000
0.511	0.097	0.294	0.000	0.012	0.026	0.416	0.000
0.189	0.077	0.054	0.979	0.531	0.749	0.726	0.000
0.800	0.068	0.087	0.051	0.000	0.013	0.237	0.000
0.431	0.968	0.514	0.036	0.164	0.871	0.857	0.000
0.202	0.900	0.677	0.000	0.001	0.011	0.308	0.000
0.331	0.000	0.004	0.001	0.000	0.016	0.025	0.000
0.007	0.000	0.000	0.006	0.008	0.015	0.005	0.000
0.000	0.000	0.000	0.061	0.019	0.014	0.001	0.000
0.006	0.000	0.000	0.026	0.010	0.009	0.002	0.000
0.004	0.057	0.002	0.725	0.515	0.065	0.054	0.000
0.000	0.016	0.000	0.701	0.347	0.243	0.023	0.000
0.016	0.000	0.000	0.019	0.008	0.014	0.003	0.000
0.000	0.000	0.000	0.046	0.010	0.009	0.001	0.000
0.701	0.019	0.046	0.000	0.000	0.000	0.042	0.000
0.347	0.008	0.010	0.000	0.000	0.000	0.065	0.000
0.243	0.014	0.009	0.000	0.000	0.000	0.001	0.000
0.023	0.003	0.001	0.042	0.065	0.001	0.000	0.000
0.246	0.000	0.002	0.000	0.000	0.000	0.004	0.000
0.547	0.561	0.797	0.058	0.915	0.157	0.100	0.000
0.746	0.345	0.436	0.080	0.001	0.004	0.163	0.000
0.830	0.932	0.873	0.128	0.114	0.356	0.147	0.000
0.874	0.845	0.977	0.008	0.002	0.366	0.083	0.000
0.641	0.021	0.069	0.004	0.003	0.001	0.002	0.000
0.621	0.212	0.440	0.001	0.105	0.004	0.053	0.000
0.766	0.543	0.659	0.441	0.062	0.050	0.042	0.000
0.937	0.908	0.927	0.143	0.012	0.822	0.274	0.000
0.697	0.836	0.945	0.027	0.050	0.871	0.096	0.000
0.594	0.021	0.077	0.136	0.221	0.044	0.001	0.000

MgU	NUtE	PUtE	KUtE	CaUtE	MgUtE	NHI
0.000	0.009	0.001	0.508	0.571	0.000	0.000
0.967	0.000	0.020	0.000	0.000	0.007	0.000
0.000	0.882	0.100	0.052	0.057	0.013	0.189
0.002	0.001	0.151	0.249	0.064	0.024	0.000
0.656	0.291	0.528	0.614	0.815	0.632	0.347
0.000	0.894	0.000	0.670	0.148	0.001	0.209
0.014	0.325	0.250	0.015	0.187	0.013	0.044
0.001	0.266	0.004	0.226	0.002	0.012	0.006
0.000	0.510	0.008	0.131	0.129	0.000	0.083
0.001	0.780	0.724	0.470	0.369	0.050	0.326
0.004	0.867	0.603	0.887	0.967	0.093	0.519
0.001	0.651	0.431	0.927	0.908	0.052	0.310
0.792	0.610	0.599	0.321	0.696	0.337	0.439
0.246	0.547	0.746	0.830	0.874	0.641	0.621
0.000	0.561	0.345	0.932	0.845	0.021	0.212
0.002	0.797	0.436	0.873	0.977	0.069	0.440
0.000	0.058	0.080	0.128	0.008	0.004	0.001
0.000	0.915	0.001	0.114	0.002	0.003	0.105
0.000	0.157	0.004	0.356	0.366	0.001	0.004
0.004	0.100	0.163	0.147	0.083	0.002	0.053
0.000	0.199	0.001	0.380	0.111	0.000	0.005
0.199	0.000	0.016	0.003	0.014	0.001	0.000
0.001	0.016	0.000	0.088	0.736	0.000	0.001
0.380	0.003	0.088	0.000	0.000	0.175	0.060
0.111	0.014	0.736	0.000	0.000	0.436	0.351
0.000	0.001	0.000	0.175	0.436	0.000	0.000
0.005	0.000	0.001	0.060	0.351	0.000	0.000
0.020	0.003	0.000	0.009	0.067	0.000	0.001
0.262	0.001	0.577	0.000	0.000	0.365	0.051
0.296	0.228	0.860	0.000	0.000	0.616	0.928
0.001	0.000	0.001	0.029	0.022	0.000	0.000

PHI	KHI	CaHI	MgHI
0.007	0.682	0.781	0.000
0.000	0.000	0.001	0.000
0.309	0.082	0.144	0.117
0.477	0.403	0.010	0.168
0.327	0.729	0.980	0.693
0.000	0.086	0.406	0.071
0.240	0.065	0.088	0.039
0.167	0.192	0.009	0.312
0.037	0.085	0.243	0.001
0.960	0.484	0.448	0.071
0.838	0.940	0.960	0.097
0.705	0.949	0.926	0.060
0.265	0.800	0.601	0.213
0.766	0.937	0.697	0.594
0.543	0.908	0.836	0.021
0.659	0.927	0.945	0.077
0.441	0.143	0.027	0.136
0.062	0.012	0.050	0.221
0.050	0.822	0.871	0.044
0.042	0.274	0.096	0.001
0.020	0.262	0.296	0.001
0.003	0.001	0.228	0.000
0.000	0.577	0.860	0.001
0.009	0.000	0.000	0.029
0.067	0.000	0.000	0.022
0.000	0.365	0.616	0.000
0.001	0.051	0.928	0.000
0.000	0.103	0.125	0.000
0.103	0.000	0.000	0.033
0.125	0.000	0.000	0.094
0.000	0.033	0.094	0.000
